# Risk Factors for premature birth in a hospital**^1^**

**DOI:** 10.1590/1518-8345.0775.2750

**Published:** 2016-07-25

**Authors:** Margarita E. Ahumada-Barrios, German F. Alvarado

**Affiliations:** 2 RN, Assistant Professor, Facultad de Ciencias de la Salud. Universidad Católica Sedes Sapientiae. Lima, Peru. Sanidad de la Escuela de Supervivencia en el Mar. Fuerza Aérea del Perú, Lima, Peru.; 3 PhD, Associate Professor, Facultad de Ciencias de la Salud. Universidad Católica Sedes Sapientiae. Lima, Peru. Associate Professor, Facultad de Salud Pública y Administración "CVL". Universidad Peruana Cayetano Heredia, Lima, Peru.

**Keywords:** Prematurity, Pregnancy Gemelar, Preeclampsia, Preterm Birth

## Abstract

**Objective:**

to determine the risk factors for premature birth.

**Methods:**

retrospective case-control study of 600 pregnant women assisted in a hospital, with 298 pregnant women in the case group (who gave birth prematurely <37 weeks) and 302 pregnant women who gave birth to a full-term newborn in the control group. Stata software version 12.2 was used. The Chi-square test was used in bivariate analysis and logistic regression was used in multivariate analysis, from which Odds Ratios (OR) and Confidence Intervals (CI) of 95% were derived.

**Results:**

risk factors associated with premature birth were current twin pregnancy (adjusted OR= 2.4; p= 0.02), inadequate prenatal care (< 6 controls) (adjusted OR= 3.2; p <0.001), absent prenatal care (adjusted OR= 3.0; p <0.001), history of premature birth (adjusted OR= 3.7; p <0.001) and preeclampsia (adjusted OR= 1.9; p= 0.005).

**Conclusion:**

history of premature birth, preeclampsia, not receiving prenatal care and receiving inadequate prenatal care were risk factors for premature birth.

## Introduction

World Health Organization (WHO) defined premature birth or preterm birth as the birth occurring after 20 weeks and before 37 weeks of gestation^1^. Premature birth is a syndrome associated with neonatal morbidity, which has adverse consequences for long-term health^2^ and the sum of complications during the lives of premature infants causes high neonatal mortality rates^3^. 

Premature birth has been associated with several factors, such as history of preterm birth^4^ ^-^ ^7^, anemia^8^ ^-^ ^9^, high catecholamine levels in the maternal urine^10^, tobacco consumption^11^ ^-^ ^12^, premature rupture of membranes (PROM)^5^ ^,^ ^13^, high blood pressure (HBP)^14^, vaginal bleeding^5^, intergestational intervals ≤ 1 year^5^, urinary tract infection (UTI)^5^ ^-^ ^6^ ^,^ ^15^, lack of prenatal care^13^, inadequate prenatal care^13^ ^,^ ^16^, maternal age less than 20 years^16^, maternal age over 35 years^15^ ^,^ ^17^, oligohydramnios^6^, history of induced abortion^18^ ^-^ ^20^, preeclampsia^6^ ^-^ ^7^ ^,^ ^13^ ^,^ ^21^, twin pregnancy^6^ ^-^ ^7^ ^,^ ^13^, advanced maternal age^6^.

Moreover, although there are several risk factors associated with premature birth, its etiology has not been fully determined^9^ ^,^ ^15^. There are studies on the subject in Latin America, and several of them show methodological limitations, so that this fact has prompted the need for carrying out this study in a hospital in North Lima and, thereby, contribute to the knowledge on this subject. The main objective of this study is to determine the risk factors for premature birth in a hospital in North Lima.

## Methods

**Figure 1:**
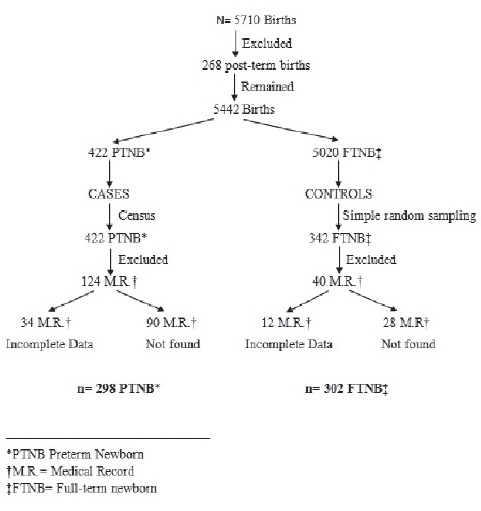
Flowchart of participation

A retrospective unmatched case-control study was carried out with a sample of 600 babies born alive at the National Hospital Sergio E. Bernales (NHSEB), located in the municipality of Lima, Peru. They were born between January 1 and December 31, 2011, with which two groups were formed:

- A case group (premature babies), consisted of 298 newborns with gestational age (GA) less than 37 weeks at birth, out of a total of 422 premature babies born during the study period. Here, 34 medical records were excluded due to incomplete data (gestational age, parity, number of prenatal controls and hemoglobin) and 90 medical records that were not found in the Archive Department of the NHSEB (as shown in Figure 1). A survey was performed for this group, considering all medical records (of mothers who gave birth prematurely in 2011), which were likely to be found at the Archive Department of the NHSEB.

- A group of unmatched controls (full-term newborns), composed of 302 newborns with a GA greater than or equal to 37 weeks and less than or equal to 42 weeks, out of a total of 342 full-term newborns, selected through simple random sampling during the study period, from a total of non-cases (N=5020). Here, 12 medical records were excluded due to incomplete data (gestational age, parity, number of prenatal controls and hemoglobin) and 28 medical records that were not found in the Archive Department of the NHSB. Simple random sampling was performed using RANDBETWEEN Function(n, N) in Excel.

It is worth mentioning that 268 medical records belonging to mothers who gave birth to a post term newborn were excluded. To determine the GA, it was taken into account the age indicated in the record book of children born in 2011, of the Neonatology service of the NHSEB, confirmed by physical examination of the newborn or Capurro method, which was registered in each medical record.

The following data were obtained from medical records: maternal age, marital status, gestational age, number of prenatal controls (appropriate, inappropriate and absent)^22^, number of abortions, number of births (vaginal and cesarean section), history of premature birth, anemia (<11 mg/dL), UTI, PROM, preeclampsia, oligohydramnios, smoking (active smoking mothers), current twin pregnancy and vaginal bleeding (bleeding during the first and second trimester of pregnancy). These data were entered into a Microsoft Excel^(r)^ 2010 database.

Stata version 12.2 was used for data processing. In univariate analysis, proportions and measures of central tendency and dispersion were calculated according to the variable type.

In bivariate analysis, the Chi-square test was used to compare two categorical variables, based on previous specific assumptions, producing crude Odds Ratios and 95% Confidence Intervals (CI); and logistic regression was used in multivariable analysis, considering all the variables with p <0.20 in the bivariate analysis. Adjusted Odds Ratios (OR) and 95% Confidence Intervals (CI) were estimated and Akaike and Bayesian information criteria were used for modelling; model goodness of fit was checked using the Hosmer-Lemeshow test. It was considered a level of significance p <0.05.

Ethics Committee of the University and the Hospital approved the study. 

## Results

During the study period, 5,710 births were registered in the National Hospital Sergio E. Bernales, where the prevalence of premature birth in the population was 7.4%. It was observed that the average age was 26.2 years, in a sample of 600 pregnant women.

According to Table 1 (bivariate analysis), the factors considered statistically significant for prematurity were: previous abortion (p = 0.04), inadequate (<6 controls) or lack of prenatal care (no control) (p <0.001), history of premature birth (p <0.001), preeclampsia (p= <.001) and bleeding (p = 0.004). 

As for the other factors investigated (age, parity, current twin pregnancy, anemia, UTI, PROM, oligohydramnios and smoking), no significant differences were found between the two groups.

**Table 1:** Bivariate analysis in pregnant women for current premature birth in a Hospital of North Lima, Peru, 2011

Risk factor	Cases n (%)	Controls n (%)	p-value
Age (years)			
mean+- S.D.	25.7 ± 6.8	26.6 ± 7.3	0.15
Parity			
0 children	97 (32.6)	107 (35.4)	0.54
1 - 2 children	155 (52.0)	157 (52.0)	
≥ 3 children	46 (15.4)	38 (12.6)	
Current twin pregnancy			
No	276 (92.6)	288 (95.4)	0.16
Yes	22 (7.4)	14 (4.6)	
Previous abortion			
No	194 (65.1)	172 (57.0)	0.04*
Yes	104 (34.9)	130 (43.0)	
PNC^†^			
Adequate (>=6)	129 (43.3)	213 (70.5)	< 0.001‡
Inadequate (<6)	122 (40.9)	64 (21.2)	
Absent (=0)	47 (15.8)	25 (8.3)	
History of premature birth			
No	191 (64.1)	260 (86.1)	< 0.001‡
Yes	107 (35.9)	42 (13.9)	
Anemia			
No	101 (33.9)	126 (41.7)	0.05
Yes	197 (66.1)	176 (58.3)	
UTI^§^			
No	168 (56.4)	177 (58.6)	0.60
Yes	130 (43.6)	125 (41.4)	
PROM^||^			
No	239 (80.2)	232 (76.8)	0.30
Yes	59 (19.8)	70 (23.2)	
Preeclampsia			
No	227 (76.2)	263 (87.1)	< 0.001‡
Yes	71 (23.8)	39 (12.9)	
Oligohydramnios			
No	266 (89.3)	282 (93.4)	0.10
Yes	32 (10.7)	20 (6.6)	
Smoking			
No	287 (96.3)	295 (97.7)	0.30
Yes	11 (3.7)	7 (2.3)	
Bleeding			
No	283 (95.0)	299 (99.0)	0.004‡
Yes	15 (5.0)	3 (1.0)	

*p<0.05

†PNC= Prenatal care

‡p<0.01

§UTI= Urinary tract infection

||PROM= Premature rupture of membranes

Table 2 shows the results of multivariate analysis. The following factors emerged as statistically significant for premature birth: current twin pregnancy (adjusted OR = 2.4; p = 0.02), inadequate prenatal care (< 6 controls) (adjusted OR = 3.2; p <0.001), absent prenatal care (no control) (adjusted OR = 3.0; p <0.001), history of premature birth (adjusted OR = 3.7; p <0.001) and preeclampsia (adjusted OR = 1.9; p = 0.005). 

**Table 2:** Multivariate analysis in pregnant women for current premature birth in a Hospital of North Lima, Peru, 2011

Risk factor	Crude OR (95% CI)		Adjusted OR (95% CI)*	p-value*
Age (years)	1.0 (0.9 - 1.0)		1.0 (1.0 -1.1)	0.03†
Parity				
0 children	1.0		-	
1 - 2 children	1.1 (0.8 - 1.6)			
≥ 3 children	1.3 (0.8 - 2.2)			
Current twin pregnancy				
No	1.0		1.0	
Yes	1.6 (0.8 - 3.3)		2.4 (1.1 - 5.1)	0.02†
Previous abortions				
No abortion	1.0		1.0	
Abortion	0.7 (0.5 - 1.0)		0.7 (0.5 - 1.0)	0.08
PNC^‡^				
Normal (>=6)	1.0		1.0	
Inadequate (<6)	3.2 (2.2 - 4.6)		3.2 (2.1 - 4.7)	< 0.001§
Absent	3.1 (1.8 - 5.3)		3.0 (1.7 - 5.4)	< 0.001§
History of premature birth				
No	1.0-		1.0	
Yes	3.5 (2.3 - 5.2)		3.7 (2.3 - 5.8)	< 0.001§
Anemia				
No	1.0		1.0	
Yes	1.4 (1.0 - 2.0)		1.2 (0.8 - 1.7)	0.47
UTI^||^				
No	1.0-		-	
Yes	1.1 (0.8 - 1.5)			
PROM^¶^				
No	1.0-		-	
Yes	0.8 (0.6 - 1.2)			
Preeclampsia				
No	1.0		1.0	
Yes	2.1 (1.4 - 3.2)		1.9 (1.2 - 3.1)	0.005§
Oligohydramnios				
No	1.0		1.0	
Yes	1.7 (1.0 - 3.0)		1.8 (0.9 - 3.4)	0.09
Smoking				
No	1.0		-	
Yes	1.6 (0.6 - 4.2)			
Bleeding				
No	1.0		1.0	
Yes	5.3 (1.5 - 18.4)		3.0 (0.8 - 11.5)	0.11

*From logistic regression

†p<0.05

‡PNC= Prenatal care

§p<0.01

||UTI= Urinary tract infection

¶PROM= Premature rupture of membranes

## Discussion

The most important findings of this study are: a) the prevalence of premature birth was 7.4% and b) risk factors for prematurity were current twin pregnancy, history of premature birth, preeclampsia, inadequate prenatal care and lack of prenatal care.

Before starting further discussion, the main limitations shall be mentioned: the limitation regarding the sample is a possible selection bias, since 124 medical records were excluded from the cases group (34 medical records due to incomplete data and 90 medical records because they were not found in the Archive Department of the hospital). In the control group, 40 medical records were excluded (12 medical records due to incomplete data and 28 medical records that were not found in the Archive Department of the hospital). However, there is no evidence that the associations investigated are different in the samples not included in the cases and controls. Moreover, in the evaluation of the variables, it was not possible to measure the levels of catecholamines or antecedents of HBP, because this was a retrospective study and such data were not registered in the medical records.

This study has as strengths the retrospective and unmatched design type of cases and controls with appropriate statistical analysis. In addition, it shows a representative sample of the study population, since it was used a census in the cases group, whereas a simple random sampling was used in the control group. 

On the other hand, this work is very important because there are scarce studies in Latin America on premature birth. 

Amongst the factors investigated in this study, preeclampsia was a risk factor for premature birth^23^. According to the study of García et al., premature births were more frequent among pregnant women with preeclampsia (p <0.001, RR=5.5; 95% CI for RR: 3.7-7.1)^21^ Similarly, the study of Osorno and colleagues reported that women with preeclampsia have risk of premature birth between 1.4 and 1.9^6^. On the other hand, current twin pregnancy represents a risk factor for premature birth, which is corroborated by a Mexican study that reported that women with triple pregnancy are 40.7 times more likely to have a premature birth, whereas twin pregnancy has an increased probability of 12 times^6^. These results were very similar to those of Gene Barrios, who reported that twin pregnancy is a risk factor for this multifactorial syndrome (OR = 15.1)^7^, as demonstrated by Diaz, which also pointed out multiple pregnancy as a factor significantly associated with the occurrence of premature birth (OR = 6.2; p <0.01)^13^.

As for the history of premature birth, this showed to be a risk factor for premature birth in a subsequent pregnancy, similar to the results reported by Gene Barrios, which also reported that prior premature birth is a risk factor for prematurity (OR = 3.4; 95% CI: 1.0-12.8)^7^. Additionally, a study conducted in Indonesia showed that history of premature birth is a factor associated with premature birth in rural areas^4^. Furthermore, in two studies carried out in Mexico, premature birth was also associated with subsequent premature births^5^ ^-^ ^6^, i.e. a previous premature birth increases the risk of a second birth under the same condition.

Another risk factor for premature birth was not receiving PNC or receiving an inadequate PNC, which is supported by the study of Sánchez and coworkers, who also indicated that the lack of prenatal care is related to prematurity, otherwise it would not be possible to diagnose complications of pregnancy and provide appropriate treatment^24^.

In addition, a study of Osorno and colleagues reported that the prevalence of prematurity is higher when there is less than 6 prenatal controls (OR: 1.7; 95% CI: 1.6-2.0), whereas in women without any prenatal control, the risk is 2.3 times higher (OR: 2.3; 95% CI: 2.0-2.8)^15^.; data similar to those of a Mexican study that reported that an inadequate prenatal care increased the risk (OR = 2.0; 95% CI: 1.5-2.7)^16^. Finally, the study of Ouattara and colleagues reported that inadequate prenatal care is associated with prematurity (OR: 4.9; 95% CI: 3.0-8.0)^13^. 

It is known that premature birth remains one of the most common problems in the Peruvian perinatology despite medical advances, as verified in the study hospital in North Lima, where the prevalence of prematurity in 2011 was 7,4% in every 100 births. This rate seems higher than the observed in previous years (6.5% in every 100 births in 2010, and 6.6% in every 100 born in 2009)^25^, representing a challenge for physicians and neonatologist nurses in the prevention and care.

Therefore, this study is highly relevant to public health because the early detection and control of the risks mentioned above could result in a reduction in prematurity.

In future research, those interested in this subject are invited to conduct cohort studies, in which catecholamine levels could be measured. 

## Conclusions

The prevalence of premature birth was 7.4%. It was concluded that the risk factors for prematurity are not receiving prenatal care, receiving inadequate prenatal care, preeclampsia and history of premature birth.
